# Objectively Measured Total and Occupational Sedentary Time in Three Work Settings

**DOI:** 10.1371/journal.pone.0149951

**Published:** 2016-03-03

**Authors:** Paula van Dommelen, Jennifer K. Coffeng, Hidde P. van der Ploeg, Allard J. van der Beek, Cécile R. L. Boot, Ingrid J. M. Hendriksen

**Affiliations:** 1 Department of Life Style, Netherlands Organisation for Applied Scientific Research TNO, Leiden, The Netherlands; 2 Department of Public and Occupational Health, EMGO+ Institute for Health and Care Research, VU University Medical Center (VUmc), Amsterdam, The Netherlands; 3 Body@Work TNO-VUmc, Research Center Physical Activity, Work and Health, Amsterdam, The Netherlands; 4 Sydney School of Public Health, University of Sydney, Camperdown, Australia; Geisel School of Medicine at Dartmouth College, UNITED STATES

## Abstract

**Background:**

Sedentary behaviour increases the risk for morbidity. Our primary aim is to determine the proportion and factors associated with objectively measured total and occupational sedentary time in three work settings. Secondary aim is to study the proportion of physical activity and prolonged sedentary bouts.

**Methods:**

Data were obtained using ActiGraph accelerometers from employees of: 1) a financial service provider (n = 49 men, 31 women), 2) two research institutes (n = 30 men, 57 women), and 3) a construction company (n = 38 men). Total (over the whole day) and occupational sedentary time, physical activity and prolonged sedentary bouts (lasting ≥30 minutes) were calculated by work setting. Linear regression analyses were performed to examine general, health and work-related factors associated with sedentary time.

**Results:**

The employees of the financial service provider and the research institutes spent 76–80% of their occupational time in sedentary behaviour, 18–20% in light intensity physical activity and 3–5% in moderate-to-vigorous intensity physical activity. Occupational time in prolonged sedentary bouts was 27–30%. Total time was less sedentary (64–70%), and had more light intensity physical activity (26–33%). The employees of the construction company spent 44% of their occupational time in sedentary behaviour, 49% in light, and 7% in moderate intensity physical activity, and spent 7% in sedentary bouts. Total time spent in sedentary behavior was 56%, 40% in light, and 4% in moderate intensity physical behaviour, and 12% in sedentary bouts. For women, low to intermediate education was the only factor that was negatively associated with occupational sedentary time.

**Conclusions:**

Sedentary behaviour is high among white-collar employees, especially in highly educated women. A relatively small proportion of sedentary time was accrued in sedentary bouts. It is recommended that worksite health promotion efforts should focus on reducing sedentary behaviour through improving light intensity physical activity.

## Introduction

Over the past fifty years, work has become increasingly sedentary [1]. Sedentary behaviours (from the Latin word sedere–‘to sit’) refer to those activities (i.e., during commuting, at work, in the domestic environment and during leisure) that require a very low energy expenditure (≤1.5 Metabolic Equivalent of Task) while sitting or reclining [2]. There has been a rapid accumulation of epidemiological studies to show that time spent sedentary, often independent of moderate-to-vigorous physical activity, is associated with premature mortality, certain cancers (i.e., colon, endometrial and lung), type 2 diabetes, obesity, and biomarkers of cardio-metabolic health [3–12]. Moreover, prolonged sedentary time or uninterrupted sedentary periods has been shown to be detrimentally associated with several cardio-metabolic health outcomes [11,12]. A meta-analysis showed that interrupting bouts of sedentary behavior with light-intensity activity might help control adiposity and postprandial glycemia [13]. An expert-based recommendation rooted in musculoskeletal health, advises to change posture (i.e., from sitting to standing or walking) after a prolonged sitting period lasting 30 minutes [14].

As approximately one third to half of our daily sitting occurs at work, occupational sitting has important occupational and public health implications [14–17]. In a Dutch cross-sectional survey, employees reported sitting on average seven hours per day, with the highest amount of sitting in the information technology, banking and insurance sectors and the lowest amount in the construction, health care and catering sectors [18]. Comparison across gender in sedentary behaviour is limited. Recently, a study using data from 32 European countries showed that men reported higher total weekly sitting time compared to women [19].

Generally, however, objective data on sedentary behaviour is lacking within the work context. Most of the results are based on self-reported data [20–23], which is prone to recall and social desirability bias [24–25]. Accelerometers (activity monitors) are commonly used to provide an objective measure of sedentary time and physical activity [26]. Accelerometers can assess sedentary, light, moderate and vigorous intensity physical activity, and bouts of prolonged sedentary time (≥30 minutes).

Thus far, there is no strong evidence for the associations between sedentary time with work-related factors, such as work vitality, job satisfaction, work performance, and sickness absenteeism. It is, however, likely that prolonged sedentary time leads to detrimental work-related outcomes, potentially through the pathway of developing adverse health effects.

In the context of detrimental effects of a sedentary lifestyle, there is a need to increase our understanding of patterns of sedentary behaviour and to clarify the association between work setting and sedentary time. Hence, our primary aim was to determine the proportion of objectively measured total and occupational sedentary time in three different work settings, and to determine factors associated with total and occupational sedentary time. A secondary aim was to determine the proportion of physical activity and prolonged sedentary bouts (lasting ≥30 minutes). This aim was achieved by data from ActiGraph accelerometers from employees of a financial service provider, a research institutes, and a construction company.

## Materials and Methods

### Study population

This cross-sectional study used baseline data from three intervention studies, which were part of a research program "Vitality In Practice". These studies were randomized controlled trials including a baseline measurement [27–29]. In all three studies a subsample of the employees wore an accelerometer at baseline. Each study was conducted in a different work setting: all white-collar office employees (both team leaders and their employees) from one financial service provider (n = 92), all white-collar employees from two research institutes in the governmental sector (n = 102), and all blue-collar employees (both construction workers and factory workers) from one large construction company (n = 47).

### Ethics, consent and permissions

The participants signed an informed consent. All studies were approved by the medical ethics committee of the VU University Medical Center in Amsterdam, the Netherlands.

### Measurements

#### Accelerometry

Participants wore an accelerometer (Tri-axis Acti trainer activity monitor, ActiGraph) on the right hip during a period of seven consecutive days, removing it only for water-based activities and sleeping. Participants with at least four valid days including a minimum of two working days (defined as days where the participant reported working at least three hours) with a minimum of 10 hours of wearing time per day [30] were included in the analyses. Non-wearing bouts were classified as periods of 60 consecutive minutes of zero-count per minute, with allowance for up to 2 minutes of < 100 counts per minute (cpm). Validated accelerometer thresholds were used to define: sedentary time as <100 cpm, light intensity physical activity as 100–1951 cpm, moderate intensity physical activity as 1952–5724 cpm, and vigorous intensity physical activity as ≥5725 cpm [26]. Prolonged bouts of sedentary time were defined as uninterrupted sitting periods lasting 30 minutes or more, corresponding to an earlier proposed definition [14]. Total and occupational time spent sedentary, and in light, moderate, and vigorous intensity physical activity, and in prolonged sedentary bouts were calculated as percentage of wearing time. Occupational time was derived from the participants’ diary, in which participants reported the exact times of leaving and arriving at work.

No diaries were available for the construction employees. For this group, we used the regular working hours (according to the Human Resource Departments) of 7 am until 3 pm to calculate working hours.

#### General characteristics, health- and work-related factors

Educational level was divided into low education (no education, primary school, lower vocational education or lower secondary school), intermediate education (intermediate vocational education or intermediate/higher secondary education), and high education (higher vocational education and university). Body mass index was calculated as the body weight in kilograms divided by the square of the body height in meters (kg/m^2^). Body weight and body height were assessed by occupational physicians or research assistants, except for the participants of the financial service provider, for whom it was self-reported. Body mass index was dichotomized: overweight (≥25 kg/m^2^) and no overweight (<25 kg/m^2^) following the World Health Organization definitions [31]. Self-reported health status was measured by one item: “In general, would you say your health is?” on a 5-point scale, (1 = poor to 5 = excellent) from the Dutch validated version of the Research and Development-36 [32]. Work vitality was measured using the vigour scale (i.e., 6 items) from the Utrecht Work Engagement Scale [33]. For example, “At work I feel full of energy” or “When I work I feel strong and fit”. The items were measured on a 7-point scale from “never” to “always”, and categorized into low (1–3.5) and high (3.6–7). The psychometric properties of this scale have been tested previously and results have indicated an acceptable reliability of the vigour scale (α = 0.83) [34]. Job satisfaction was assessed using one overall question on a 5-point rating scale from”highly dissatisfied” to “very satisfied”, i.e., “To what extent are you satisfied with your job”? A single-item measure of job satisfaction has been found to correlate highly with job satisfaction scales, and was, therefore, considered valid [35,36]. Work performance was assessed with the Netherlands working conditions survey scale that uses three items on a 5-point scale (i.e., “agree” to “don’t agree”) from; e.g., “I perform well in my work” [37]. Baseline sickness absence data were collected from company records over the previous year. Sickness absenteeism was subsequently categorized into 0 days, 1–7 days, and >7 days.

#### Statistical analysis

Descriptive analyses were performed to summarize the characteristics of the employees of the three work settings with means and standard deviations (SD) or percentages. First, we averaged the proportion of sedentary time, light intensity physical activity, moderate physical activity, and vigorous physical activity, sedentary bouts for total time and occupational time over all days within each person. Second, we calculated the group means, stratified by gender. Linear regression analyses were performed to compare the averaged proportion of sedentary time, sedentary bouts and the different physical activity intensity levels between work settings, total and occupational time, and between men and women. Multivariate linear regression analyses were performed to examine the associations of age, educational level, overweight, self-rated health, work vitality, job satisfaction, work performance, and sickness absenteeism with total and occupational averaged proportion of sedentary time, adjusted for work setting. Only male employees were available in the construction company. Therefore, all analyses were stratified by gender to allow a fair comparison between the construction company and the other two work settings. Furthermore, none of the employees in the construction company were highly educated, and, therefore, we did not include educational level in the model based on all male employees.

All variables were checked for normality using Q-Q plots (a graphical method to compare two probability distributions). In case of non-normality, a log transformation was applied.

All analyses were performed in SPSS 20.0 (SPP, Inc., Chicago Illinois) and accelerometer data were analyzed using the ActiLife 3.2.2 software. Significance was set at p<0.05 (two-sided).

## Results

### General characteristics of the three work settings

Accelerometer data were available for 92 employees from the financial service provider, 102 employees from the research institutes, and 47 employees from the construction company. No diaries on occupational time were available for 7 employees of the financial service provider and 2 employees of the research institutes, and these employees were excluded. After selecting employees who wore the accelerometer at least four valid days including a minimum of two working days (defined as days where the employees reported working at least three hours) with a minimum of 10 hours of wearing time per day, in total 80 employees from the financial service provider, 87 employees from the research institutes, and 38 employees from the construction company were available for analyses (see Table 1). All characteristics differed significantly between two or more work settings, except for sickness absenteeism and job satisfaction. Compared to the financial service provider, employees from the research institutes were more likely to be women, older and more likely to have attained a higher vocational or university degree. All construction employees were men and none of them were highly educated. Compared to the other two work settings, overweight was more present (p<0.001), and work performance was lower (p<0.002) among the employees of the construction company.

**Table 1:** Characteristics of employees of the three work settings.

	Financial service provider	Research institutes	Construction company
	n = 80	n = 87	n = 38
Gender [n (%)] ^*$			
Men	49 (61.3)	30 (34.5)	38 (100)
Women	31 (38.8)	57 (65.5)	0 (0)
Age (years) [mean (SD^b^)] ^$	42.8 (10.1)	47.4 (9.3)	48.9 (9.1)
Missing	0	0	5
Educational level [n (%)]^a^ ^*$			
Low	3 (3.8)	0 (0.0)	21 (67.7)
Intermediate	30 (37.5)	21 (24.1)	10 (32.3)
High	47 (58.8)	66 (75.9)	0 (0.0)
Missing	0	0	7
Overweight [n (%)] ^*			
No	46 (57.5)	50 (57.5)	5 (16.1)
Yes	34 (42.5)	37 (42.5)	26 (83.9)
Missing	0	0	7
Self-reported health status [n (%)]*			
Poor/low	12 (15.6)	3 (3.4)	2 (6.1)
Good	40 (51.9)	43 (49.4)	23 (69.7)
Very good/ excellent	25 (32.5)	41 (47.1)	8 (24.2)
Missing	0	0	5
Work vitality [n (%)] $			
Low (1–3.5)	4 (5.1)	24 (27.6)	4 (12.5)
High (3.6–7)	75 (94.9)	63 (72.4)	28 (87.5)
Missing	0	0	6
Sickness absenteeism (days) [n (%)]			
0	29 (39.2)	37 (47.4)	12 (36.4)
1–7	23 (31.1)	24 (30.8)	8 (24.2)
>7	22 (29.7)	17 (21.8)	13 (39.4)
Missing	6	9	5
Job satisfaction [n (%)]			
Low (1–2)	13 (16.2)	9 (10.3)	2 (6.5)
Neutral (3)	54 (67.5)	58 (66.7)	21 (67.7)
High (4–5)	13 (16.2)	20 (23.0)	8 (25.8)
Missing	0	0	7
Log of work performance+ [mean (SD^b^)] ^*(1 = low—5 = high, without the log)	1.9 (0.7)	1.8 (0.5)	1.4 (0.5)
Missing	0	0	6

^a^Low education (no education, primary school, lower vocational education or lower secondary school), intermediate education (intermediate vocational education or intermediate/higher secondary education) and high education (higher vocational education and university)

^b^SD = Standard Deviation

^^^ Significant difference between construction company and financial service provider

* Significant difference between construction company and research institutes

^$^ Significant difference between research institutes and financial service provider

For testing, educational level was categorized into high versus low/intermediate, because of the small number of low educated employees of the research institutes and financial service provider. Similarly, self-rated health was categorized into poor/low/good versus very good/excellent.

^+^Because of non-normality, the log of work performance was used for testing

### Sedentary time, physical activity and prolonged sedentary bouts

Table 2 reports the results of total and occupational sedentary time, physical activity and prolonged sedentary bouts among the three work settings, separately for men and women. Total mean wearing time per day varied between the three settings from 14.7 to 15.4 hours, whereas this was 7.7 to 8.5 hours during occupational time (i.e. 50–57% of total time). The interquartile range (IQR) of total wearing time was (14.3–15.5) for employees of the financial service provider and the research institutes and (14.8–16.4) for employees of the construction company, and IQR of total wearing time during occupation was (7.6–8.9) for employees of the financial service provider and the research institutes and (7.6–8.0) for employees of the construction company. Most results differed significantly between the construction company and the other two work settings. The employees of the financial service provider and the research institutes mostly spent their occupational time in sedentary behaviour (76–80%), 18–20% in light intensity physical activity, 3–5% in moderate to vigorous intensity physical activity, and 27–30% in sedentary bouts. In total time, they were less sedentary (64–70%) (p<0.001), and had more light intensity physical activity (26–33%) (p<0.001) compared to occupational time. The employees of the construction company spent less occupational time in sedentary (44%) (p<0.001), engaged more in light (49%) (p<0.001) and moderate intensity physical activity (7%) (p<0.003), and spent less time in sedentary bouts (7%) (p<0.001) compared to the other two work settings. In total time, they were more sedentary (56%) (p<0.001), had less light (40%) (p = 0.001) and moderate (4%) (p = 0.003) intensity physical activity, and spent more time in sedentary bouts (12%) (p = 0.02) compared to occupational time. Occupational time spent in moderate to vigorous intensity physical activity was more dispersed among construction workers (SD = 5.2) compared to the other two work settings (SD = 3.1 and 2.2).

**Table 2:** Total and occupational time in sedentary behaviour, physical activity and prolonged sedentary bouts.

	Total Time [mean (SD)]			Occupational Time [mean (SD)]		
	Financial service provider	Research institutes	Construction company	Financial service provider	Research institutes	Construction company
	n = 80	n = 87	n = 38	n = 80	n = 87	n = 38
Total wearing time (hrs/day)						
Men	14.9 (1.1)	15.0 (0.8)	15.4 (1.2)^	8.5 (1.0)	8.2 (1.1)	7.7 (0.7)^
Women	14.7 (1.0)	14.8 (0.8)		8.3 (1.0)	7.8 (1.2)$	
% in sedentary time						
Men	70.0 (5.2)	65.7 (5.3)$	55.5 (9.3)*^	78.5 (5.6)+	77.0 (7.4)+	43.6 (16.9)*^+
Women	67.4 (6.9) ~	63.5 (6.9$		79.5 (5.9)+	76.3 (7.6)$+	
% time in light intensity physical activity						
Men	26.0 (5.2)	29.9 (5.1)$	40.2 (8.4)*^	17.7 (5.2)+	18.3 (6.0)+	49.2 (14.2) *^+
Women	29.2 (6.6) ~	32.8 (6.4)$~		17.6 (5.4)+	20.4 (7.1)+	
% in time in moderate intensity physical activity						
Men	3.6 (1.1)	4.0 (2.2)	4.2 (2.4)	3.5 (2.0)	4.1 (2.2)	7.1 (5.2) *^+
Women	3.1 (1.2)~	3.5 (1.9)		2.4 (1.4)~	3.2 (1.9)~	
% time in vigorous intensity physical activity						
Men	0.3 (0.5)	0.4 (0.8)	0.1 (0.3)^	0.3 (0.9)	0.5 (2.2)	0.0 (0.0) *^
Women	0.3 (0.6)	0.2 (0.4)		0.4 (1.1)	0.1 (0.4)	
% in moderate to vigorous intensity physical activity (MVPA)						
Men	4.0 (1.3)	4.4 (2.3)	4.3 (2.4)	3.8 (2.2)	4.7 (3.1)	7.2 (5.2)^+
Women	3.4 (1.4)	3.7 (2.0)		2.8 (1.9)~	3.3 (2.0)~	
% time in sedentary bouts ≥30 minutes						
Men	22.3 (8.9)	22.2 (6.9)	12.2 (7.1)*^	27.4 (16.3)	30.0 (14.9)+	7.2 (10.7)*^+
Women	21.9 (11.2)	19.2 (7.6)		29.8 (17.9)+	28.3 (15.1)+	

^^^ Significant difference between construction company and financial service provider

* Significant difference between construction company and research institutes

^$^ Significant difference between research institutes and financial service provider

^+^Significant difference between total time and occupational time within this group (total wearing time was not tested)

^~^Significant difference between men and women within this group

For all three work settings, the proportion of vigorous physical activity was very low (0.0–0.5%). Compared to women, men spent more time in moderate physical activity in occupational time (p<0.05) and in total time (p = 0.04, only significant in employees of the financial service provider), while women spent more time in light intensity physical activity than men (p<0.05).

### Associations with sedentary time

Table 3 present the associations of general characteristics, health- and work-related factors with total and occupational sedentary time. The upper part of Table 3 reports the findings for men within the three work settings, where no statistically significant associations were found. Among male employees of the financial service provider and the research institutes only, level of education was not statistically significantly associated with total and occupational time. The lower part of Table 3 reports the results for women. The table shows that low to intermediate education was the only factor that was negatively associated with occupational sedentary time (p = 0.009). Mean occupational sedentary time was 73.7% (SD = 7.43) for women with a low to intermediate education, whereas this was 78.8% (SD = 6.59) for women with a high education. Moreover, less time in sedentary bouts were reported in low to intermediate educated women compared to highly educated women (23.3% versus 30.9%, p = 0.045, not shown in table).

**Table 3:** Associations of general characteristics, health- and work-related factors with sedentary time in men (n = 102) and women (n = 76).

		Total sedentary time (%)	Occupational sedentary time (%)
	n	Adj. B (95%CI)^	Adj. B (95%CI)^
**men**			
Age (years)	102	0.03 (-0.13,0.18)	-0.02 (-0.26,0.22)
Overweight			
No	40	-1.25 (-4.30,1.79)	-1.63 (-6.34,3.08)
Yes	62	ref	ref
Self-reported health status			
Poor/Low/good	66	1.88 (-1.22,4.97)	2.66 (-2.12,7.45)
Very good/excellent	36	ref	ref
Work vitality			
Low (1–3.5)	16	-3.38 (-7.74,0.97)	-3.85 (-10.6,2.90)
High (3.6–7)	86	ref	ref
Job satisfaction			
Low/neutral	81	-2.41 (-5.94,1.12)	-5.28 (-10.7, 0.18)
High	21	ref	ref
Log of work performance+ (1 = low—5 = high, without the log)	102	-1.63 (-11.5,8.22)	-6.19 (-21.4,9.06)
Sickness absenteeism (days)			
0	45	ref	ref
1–7	29	-0.10 (-3.57,3.37)	2.57 (-2.80,7.94)
>8	28	-1.79 (-5.36, 1.79)	-2.56 (-8.09,2.98)
**women**			
Age (years)	76	-0.06 (-0.27,0.15)	-0.11 (-0.31, 0.09)
Educational level^a^			
Low/intermediate	20	-2.30 (-6.29,1.69)	-5.13 (-8.94,-1.33)
High	56	ref	ref
Overweight			
No	51	0.05 (-4.05,4.15)	-0.54 (-4.45,3.37)
Yes	25	ref	ref
Self-reported health status			
Poor/Low/good	46	0.24 (-3.41,3.89)	1.20 (-2.28,4.68)
Very good/excellent	30	ref	ref
Work vitality			
Low (1–3.5)	13	-3.03 (-7.49,1.43)	1.28 (-2.98,5.53)
High (3.6–7)	63	ref	ref
Job satisfaction			
Low/neutral	61	0.81 (-3.80,5.42)	3.59 (-0.80,7.99)
High	15	ref	ref
Log of work performance+ (1 = low—5 = high, without the log)+	76	1.70 (-13.3,16.7)	0.78 (-13.5,15.1)
Sickness absenteeism (days)			
0	32	ref	ref
1–7	25	1.55 (-2.35,5.46)	2.09 (-1.64,5.81)
>8	19	1.84 (-2.79,6.48)	-2.58 (-7.01,1.84)

^^^Adjusted for work setting and all other factors in this model

^a^Low education (no education, primary school, lower vocational education or lower secondary school), intermediate education (intermediate vocational education or intermediate

For testing, educational level was categorized into high versus low/intermediate, because of the small number of low educated employees of the research institutes and financial service provider. Similarly, self-rated health was categorized into poor/low/good versus very good/excellent.

^+^Because of non-normality, the log of work performance was used for testing

## Discussion

The employees of the financial service provider and the research institutes spent most of their occupational time and total time in sedentary behavior; 76–80% and 64–70%, respectively. Women spent their total time more in light intensity physical activity and were less sedentary compared to men. The employees of the construction company spent less occupational time sedentary (44%) and engaged more in light intensity physical activity (49%) than the other two work settings. In total time, the employees of the construction company were more sedentary (56%) and had less light (40%) intensity physical behavior compared to their occupational time. Time spent in prolonged sedentary bouts was 27–30% for the employees of the financial service provider and the research institutes, and only 7% for the employees of the construction company. Health- and work-related factors that we included in our study were not associated with sedentary time. The only significant factor among women was educational level with highly educated women engaging more in occupational sedentary behaviour compared to women with a low to intermediate education.

In our study, we found that employees of the financial service provider and the research institutes spent most of their occupational time in sedentary behaviour. An earlier study on self-reported sedentary behaviour among Dutch employees reported that total sitting time was on average 423 minutes per day [18]. If we assume that sleeping time is on average 8 hours, total sitting time during the day was approximately 44% (= 423/((24–8)*60)) in that study [18]. Our study reports higher proportions of total time spent in sedentary behaviour (63.6–70.0%) when assessed objectively. Differences between studies may (partly) be caused by differences in objectively measured data versus self-reported data, and type of work settings included. Our results were in line with those of a Swedish study of 140 call centre employees, which revealed that objectively measured sedentary time took up 75% of a work day [38]. As was shown in the results, a relatively small amount of sedentary time was accrued in sedentary bouts, which is likely to reveal that people do stand-up during working hours regularly.

For construction employees, the opposite was found; they spent less occupational time in sedentary behaviour, less occupational time in sedentary bouts, and more occupational time in light and moderate intensity activity than employees of the other two work settings. Our findings are similar to an Australian study among three occupations (193 employees using accelerometers) showing that occupational time was mostly spent sedentary (77%) [39]. Moreover, another Australian study demonstrated that the self-reported work-related share for sitting among blue-collar employees (e.g. construction employees) was much lower, which is also in line with the present study. However, the present study showed that the construction employees spent more time in light intensities (49% vs. 21% for Australian employees), but less time in moderate physical intensities (7.1% vs. 58% for Australian employees), and vigorous physical intensities (0.0 vs. 21% for Australian employees) [40]. These large differences may be caused by differences in objectively measured data versus self-reported data.

Several general characteristics and work-related factor were collected in our study. A high proportion of construction workers were obese and reported a lower work performance than the other two work settings. This high proportion of obese workers is in agreement with data obtained from periodic health screenings among 24,294 construction workers that showed that the prevalence of overweight and obesity in construction workers is higher than in the general Dutch adult population. Of all construction workers 65% were overweight and 16% obese. [41]. Literature has also shown that construction workers are less motivated and may, therefore, have a lower work performance [42]. The examination of the association between sedentary time with general characteristics and work-related factors showed that women overall spent more of their time in light intensity physical activity and were less sedentary, which is likely to be consistent with traditional gender role patterns (e.g., housework, supporting family members). Only low to intermediate education of female employees was negatively associated with occupational sitting time, meaning that low to intermediate educated women spent less time sitting during work. Several other studies have confirmed a lower sitting time among less educated individuals, but no consistent differences were found in sitting time by gender [43]. Our results did not show significant associations between sedentary time with work-related factors, but more research with longitudinal measurements is needed to confirm this result. Even though the relationship between job satisfaction and occupational sedentary time was not significant for either gender, both analyses approached significance but in the opposite direction for each gender. Women spent more occupational time in sitting behavior when they were less satisfied with their job, while men showed more occupational time in sitting behavior when they were more satisfied.

The primary strengths of our study are that we used accelerometer data, and objective sickness absenteeism data, and we had information on employees in different work settings. The majority of previous research on occupational sedentary time used self-reported measures, while self-report is subject to bias. With accelerometers discrete periods of sitting time, physical activity and prolonged sedentary bouts can be derived to examine how sedentary time is accumulated during a workday. Another strength is that we evaluated three work settings, which gave us insight into the largest differences present between white- and blue-collar employees in total and occupational sedentary time. This information can be used to tailor interventions to specific occupational groups.

Yet, some limitations of our study have to be discussed. A limitation is the relatively low sample of employees from the construction company. Furthermore, when stratifying by gender we had a relatively low number of employees from the financial service provider and research institutes. These low sample sizes have reduced the precision of the estimates, and the lack of power to achieve statistically significant associations. Clinically relevant associations may be job satisfaction and work performance with occupational sedentary time in men, but more research is needed to confirm this result. Furthermore, the hip worn accelerometer does not provide information on body posture or the energy cost of load (e.g., manual carrying). As a result, it could be that some time standing was misclassified as sitting. Besides standing, accelerometers are less sensitive to detect biking, which is common in the Netherlands [20]. Also other devices are available, such as the activPAL (PAL technologies Limited, Glasgow, UK), which may be more accurate for measuring sitting, standing, and walking than the Actigraph. Research has shown that this device is valid and responsive [44,45]. For future research, more sophisticated technologies are recommended. Also, combining geo-location data with accelerometer data could provide more detailed information on the context of sedentary behaviour, especially outside the work setting. In our study, we used a widely used cut-off point of <100 cpm for sedentary behaviour. However, the cut-off points or activity intensity thresholds vary in the scientific literature, and a universally accepted cut-off point is not available [46]. The same applies for the selected epoch length and criteria for determining a sedentary bout. Employees from the financial service provider and the research institutes were asked to keep a diary with their activities, which may have influenced their activity behaviour. Precise data on working hours were not available for the employees of the construction company. This may have had an effect on the outcomes, although it is unknown in which direction.

Irrespective of how sedentary time is measured, it is clear that employees sit for a considerably prolonged stretch of time. Our study shows that employees engaged about one third of their total wearing time in light intensity physical activity, which is known to be beneficial. Therefore, it is recommended that worksite health promotion should be directed at improving light intensity physical activity (which includes standing), for example by introducing sit-stand desks. A positive finding of our study is that a relatively small amount of sedentary time was accrued in sedentary bouts. Studies have shown that individuals frequently interrupting sedentary time may experience positive health effects, such as lower levels of cardiometabolic risks, than those accumulating sedentary behavior with less frequent interruptions [47].

As expected, employees of the financial service provider and those of the research institutes spent more occupational time sedentary than employees of the construction company. Worksite health promotion efforts should, therefore, be directed at reducing sedentary time among these groups. However, construction employees are also exposed to the risks of a sedentary lifestyle, but in this group sitting is more likely to occur outside working hours. Hence, opportunities to intervene in sedentary behaviour in this group should be more concentrated on leisure time.

For intervention development, a social-ecological framework is recommended, in which attention is given to the individual and environmental determinants of behaviour [48]. The social-ecological model focuses on making changes to the individual, as well as to the social, political and/or organizational environment. To illustrate, sedentary behaviour may be a function of individual factors, such as beliefs, but it could also be influenced by social factors, such as social norms and environmental/political factors, such as the availability of a sit-stand workstation. The focus of future research should be on interventions embedded within a social-ecological framework.

## Conclusions

Occupational sedentary time was highly prevalent in the employees of financial service providers and research institutes, especially among highly educated women, and was much less prevalent among employees in the construction industry. Therefore, worksite health promotion efforts focussed on sedentary behaviour should be tailored to occupations and/or sectors with white-collar workers. Such efforts might also particularly focus on highly educated women, although future studies are needed to replicate this finding first. It is recommended to reduce sedentary behaviour through improving light intensity physical activity, as employees engaged only in light intensity physical activity one third of total time. Opportunities to intervene in sedentary behaviour in employees with physically demanding work should primarily focus on leisure time.
